# Bacterial Communities in Malagasy Soils with Differing Levels of Disturbance Affecting Botanical Diversity

**DOI:** 10.1371/journal.pone.0085097

**Published:** 2014-01-20

**Authors:** Leah C. Blasiak, Alex W. Schmidt, Honoré Andriamiarinoro, Temesgen Mulaw, Rado Rasolomampianina, Wendy L. Applequist, Chris Birkinshaw, Félicitée Rejo-Fienena, Porter P. Lowry, Thomas M. Schmidt, Russell T. Hill

**Affiliations:** 1 Institute of Marine and Environmental Technology, University of Maryland Center for Environmental Science, Baltimore, Maryland, United States of America; 2 The Center for Microbial Sciences, University of Michigan, Ann Arbor, Michigan, United States of America; 3 Missouri Botanical Garden, St. Louis, Missouri, United States of America; 4 Laboratoire de Microbiologie de Environnement, Centre National de Recherches sur l′Environnement, Antananarivo, Madagascar; 5 Département Systématique et Évolution (UMR 7205), Muséum National d'Histoire Naturelle, Paris, France; U.S. Geological Survey, United States of America

## Abstract

Madagascar is well-known for the exceptional biodiversity of its macro-flora and fauna, but the biodiversity of Malagasy microbial communities remains relatively unexplored. Understanding patterns of bacterial diversity in soil and their correlations with above-ground botanical diversity could influence conservation planning as well as sampling strategies to maximize access to bacterially derived natural products. We present the first detailed description of Malagasy soil bacterial communities from a targeted 16S rRNA gene survey of greater than 290,000 sequences generated using 454 pyrosequencing. Two sampling plots in each of three forest conservation areas were established to represent different levels of disturbance resulting from human impact through agriculture and selective exploitation of trees, as well as from natural impacts of cyclones. In parallel, we performed an in-depth characterization of the total vascular plant morphospecies richness within each plot. The plots representing different levels of disturbance within each forest did not differ significantly in bacterial diversity or richness. Changes in bacterial community composition were largest between forests rather than between different levels of impact within a forest. The largest difference in bacterial community composition with disturbance was observed at the Vohibe forest conservation area, and this difference was correlated with changes in both vascular plant richness and soil pH. These results provide the first survey of Malagasy soil bacterial diversity and establish a baseline of botanical diversity within important conservation areas.

## Introduction

While the effects of land-use on the biodiversity of plants and animals are well-studied, much less is known about how disturbance shapes microbial communities. Ecological studies on the effects of disturbance on plant communities have shown that smaller disturbances may increase floral species richness, while larger ones can lead to decreased richness, loss of botanical biodiversity, or even extinction of locally endemic species (reviewed in [1]). Large disturbances such as fires or conversion of native habitats to agriculture may take centuries to completely reverse, and the re-establishment of a primary or secondary plant community often follows a predictable pattern of community succession. Advances in sequencing technology, using deep pyrosequencing of 16S rRNA gene amplicons, now permit interrogation of soil bacterial community composition in sufficient detail to begin to investigate the effects of land-use disturbance on below-ground microbial diversity. Additionally, it is now possible to investigate whether impacts to above-ground plant diversity are paralleled by changes in soil bacterial diversity. Understanding the effects of land-use on soil bacterial diversity may add a new dimension to conservation goals because soil bacteria provide critical ecosystem services and are an important source of biodiversity for drug discovery, and because bacterial communities may be early indicators of ecosystem disturbance [2], [3], [4].

Recent studies have used multiplexed pyrosequencing of 16S rRNA genes and other non-culture-based methods to identify parameters that correlate with soil bacterial community diversity or composition, but their causal relationships in driving soil bacterial community structure remain unclear. Many studies have now documented the important role of soil pH, which is strongly correlated to soil bacterial diversity (richness and evenness) at local and global scales [5], [6], [7], [8]. Other soil edaphic parameters, including soil nitrogen (N) amendment [9], [10] and total N or carbon (C) [6], [11] have been found to be significantly associated with shifts in bacterial community composition. The taxonomic composition of soil bacterial communities can also be differentiated by land-use. For example, vegetation type [7], conversion of native vegetation to agriculture [12], [13], [14], and agriculture management practices [14], [15], [16] all correlate with changes in taxonomic composition, although these changes often co-vary with changes in soil chemistry. Vegetation type correlates with bacterial community composition locally within a given soil type or as a secondary factor after soil edaphic properties [7], [17], [18], [19]. These correlations with plant diversity may represent indirect interactions (covariance of biogeographic patterns in plant and microbial communities) or direct interactions through influence of plant root exudates, organic matter deposition, and/or specific interactions with plant rhizospheres. For example, bacterial isolation from roots of endemic Malagasy rosewood trees has yielded a wide diversity of root-nodulating bacteria [20].

We hypothesized that loss of plant species richness in Malagasy forests through both human and non-human impacts is correlated with a loss of bacterial diversity and a change in bacterial community composition. Several studies have documented decreases in bacterial diversity or functional diversity with different types of disturbance, including impacts from wildfires, camping, and agriculture [9], [21], [22], [23], [24]. However, grassland soils have generally been found to harbor higher bacterial diversity than forest soils, and so disturbance of forests and conversion to grasslands may actually increase soil bacterial diversity and richness [6], [13]. The most detailed study thus far on the impact of forest disturbance on microbial communities demonstrated a significant effect of timber harvesting, organic matter removal, and soil compaction on soil bacterial community composition in coniferous forests even 10–15 years after the disturbance and despite replanting with tree seedlings [3].

In this study, we aimed to quantify the changes in soil bacterial communities with differing levels of disturbance to forest plant communities in Madagascar. Madagascar is a biodiversity hotspot with significant conservation value, and the country contains an astonishing botanical diversity including an estimated 13,000–14,000 species of vascular plants of which nearly 90% are endemic to this island nation [25], [26], [27]. The study sites included three Malagasy forests that have been prioritized for conservation efforts based on work by the Missouri Botanical Garden as part of an International Biodiversity Cooperative Group grant. Within each forest, we established two 0.1 ha plots that were representative of different disturbance levels present at the site as assessed by their plant community structure. Vascular plant species richness and plant community structure, including the number and size of stems, were quantified within each plot, and the soil bacterial communities were investigated using barcoded 454 pyrosequencing of the 16S rRNA gene. This study represents the most detailed characterization to date of plant community structure and soil bacterial diversity in Malagasy forests. The resulting data provide an important initial reference for monitoring the effects of future conservation efforts within the selected forests. The observed patterns of bacterial community composition may also influence the design of sampling strategies to maximize access to bacterial taxonomic and genetic diversity for drug discovery efforts.

## Materials and Methods

### Site description

This study was conducted within three humid forests (as defined by [28]) located in eastern Madagascar: the forests of Analalava, Pointe à Larrée, and Vohibe (Table 1). All three sites are located in a per-humid climate zone [29], but while Analalava and Vohibe are located on basement rock, Pointe à Larrée is situated on old sand dunes [30]. The loose sand geology at Pointe à Larrée and the proximity of this forest to the sea result in a distinct subclass of humid forest known as littoral forest [28]. The Vohibe Forest is at a higher elevation than the other two sites. Each of the three forests is owned by the Malagasy government, and they are currently the focus of activities that will lead to their being classified and managed as new protected areas within Madagascar's protected area network. Permits for access to sampling sites and for collection of botanical and soil samples were obtained from the Malagasy Ministère de l′Environnement et des Forêts.

**Table 1:** Description of the 0.1

Site	Forest	Subjective evaluation of disturbance	Georeference (and elevation)	Known disturbance history
A1	Analalava	High	S 17° 42′ 42,3″/E 049° 27′ 18,7″ (49 m)	Subjected to selective exploitation of medium and large trees and the collection of fence posts until 2005 when the forest was protected. Impacted by Cyclone Ivan in 2008 but sustained little damage.
A2	Analalava	Moderate	S 17° 42′ 18,9″/E 049° 27′ 22,9″ (56 m)	As at A1, but with selective exploitation of mainly large trees.
P1	Pointe à Larrée	High	S 16° 47′ 14,5″/E 049° 44′ 27,3″ (15 m)	In past decades and until present this forest is subjected to the selective exploitation of large trees. The forest was impacted by Cyclone Ivan in 2008 with consequent major damage.
P2	Pointe à Larrée	Moderate	S 16° 47′ 45,5″/E 049° 44′ 38,0″ (15 m)	As at P1, but with selective exploitation of large trees now rare.
V1	Vohibe	Very high	S 19° 09′ 45,7″/E 048° 35′ 02,3″ (598 m)	Subjected to one cycle of shifting cultivation (trees cut, left to dry and burnt, and then land cultivated until soil exhausted). The last crops were cultivated on this land in about 2002.
V2	Vohibe	Very low	S 19° 09′ 32,2″/E 048° 34′ 39,1″ (646 m)	In the past possibly subjected to very rare selective exploitation of precious woods and timber.

A total of six plots of 50 m×20 m were established, with two plots at each of the three sites (Table 1). The two plots within each forest were selected subjectively to maximize the difference between the levels of disturbance, both human and natural, on the vegetation. All sites have been subject to historical and/or recent disturbance from cyclones. At Analalava and Pointe à Larrée, no areas of the forest have been completely protected from human impact; sites were thus selected that represent moderate and high levels of selective timber exploitation. In the Vohibe forest, a site that has been well protected from human disturbance was contrasted with one that was subject to a single cycle of shifting cultivation prior to 2002 and is now largely a monoculture of a tall rhizomatous herb, Madagascar Cardamom [*Aframomum angustifolium* (Sonn.) K. Schum.].

### Botanical diversity

The diversity of vascular plants within each 0.1 ha plot was characterized by botanists from the Missouri Botanical Garden (Table 2). Plants were identified by means of nested plots. Within the entire 0.1 ha plot all plant stems with diameter at breast height (DBH) ≥5 cm were identified. Additionally, within five 16 m^2^ plots, arranged at regular intervals along the longest transect of the plot, all plants with DBH between 1 cm to 5 cm and all herbs were identified. We did not attempt to identify tree seedlings or non-vascular plants. When possible, plants were identified to a formally named species, but in many cases such precise identification could not be made because the plants were infertile (i.e. neither flowering nor fruiting). In such cases, the plants were classified only to morphospecies: distinct identities defined on the basis of habit and leaf morphology. Plants could rarely be identified with confidence in the field, so voucher herbarium specimens were made and identified later in the laboratory using the botanical literature, mounted reference herbarium specimens available in the herbarium at the Parc Botanique et Zoologique de Tsimbazaza, Antananarivo, and the electronic database of Malagasy flora (http://www.tropicos.org/project/mada). Fertile specimens were preserved at the herbaria of the Missouri Botanical Garden and the Parc Botanique et Zoologique de Tsimbazaza (MO and TAN respectively). The measurements of stem diameters were also used to describe the structure of the vegetation in the plots in terms of stem basal area and stem abundance in various DBH classes.

**Table 2:** Description of plant community structure by plot.

Site	Subjective evaluation of disturbance	# of morpho-species of vascular plants	Stem basal area (m^2^/0.1 ha) by diameter size class			Number woody stems per 0.1 ha by stem diameter size class (cm)						
			≥10 cm	≥5 to <10 cm	≤5 cm	≥1-<5	≥5-<10	≥10-<20	≥20-<30	≥30-<40	≥40-<50	≥50
A1	High	214	1.26	1.01	2.30	1875	272	53	8	2	0	0
A2	Moderate	225	1.99	1.45	3.44	2338	391	96	12	2	0	0
P1	High	103	1.86	0.64	2.50	1300	180	47	14	5	1	0
P2	Moderate	89	2.44	0.76	3.20	1250	213	61	20	5	0	1
V1	Very high	32	0.74	0.17	0.91	163	40	17	4	0	2	0
V2	Very low	215	4.10	0.66	4.75	1225	179	73	23	9	2	3

### Soil sampling and chemical analysis

A total of 30 soil cores of 4 cm diameter and 10 cm depth were sampled from the six plots (3 forests ×2 impact levels ×5 soil cores). Five soil cores were taken in 10 m intervals along the central length of each 50×20 m plot. Soil samples were collected from Analalava and Pointe à Larrée in September 2011 and from Vohibe in October 2011, and were transported on ice to the Centre National de Recherches sur l′Environnement (CNRE) laboratory in Antananarivo, Madagascar, and stored at −20°C until processing. Soil cores were homogenized and sieved through a 2 mm mesh. Soil pH, C, N, and C:N ratio (CN) were determined at FOFIFA, the Centre National de la Recherche Appliquée au Développement Rural in Madagascar (Table S1). Briefly, soil pH was measured as a water/soil suspension, total organic carbon (C) was determined according to the ANNE method [31], and the total N by the Kjeldahl method [32].

### DNA extraction and pyrosequencing

DNA was extracted from 0.25 g of soil from homogenized soil cores using the PowerSoil DNA isolation kit (Mo Bio, Carlsbad, CA) according to the manufacturer's instructions, except that the DNA yield was increased by pooling the DNA from two DNA extraction kits for each sample. A total of 60 DNA samples were prepared (two technical replicates per soil core ×5 cores per plot ×6 plots). DNA samples were dried at 50°C immediately after extraction at the CNRE laboratory and shipped dry at room temperature to Michigan for sequencing. The V4–V6 region of the 16S rRNA gene was PCR amplified in triplicate using the following barcoded primers: forward primer 518F  =  CCAGCAGCYGCGGTAAN, forward primer adapter  =  CCTATCCCCTGTGTGCCTTGGCAGTCTCAG, reverse primer 1046R  =  CGACRRCCATGCANCACCT, reverse primer adapter  =  CCATCTCATCCCTGCGTGTCTCCGACTCAG (http://www.hmpdacc.org/doc/16S_Sequencing_SOP_4.2.2.pdf) [33]. The PCR products were then purified using Agencourt® AMPure® XP beads (Beckman Coulter, Brea, CA) and the Life Technologies Dynamag-2 Magnet. DNA concentrations were quantified and samples were combined into an equimolar pool and sequenced using a Roche GS Junior 454 Sequencer. Sequences and associated metadata were deposited in the MG-RAST database and are publically accessible (http://metagenomics.anl.gov/linkin.cgi?project=6283) with accession numbers 4538937.3–4538996.3.

### Sequence processing

The V4–V6 region of the 16S rRNA gene was sequenced from 60 soil samples (2 technical replicates ×5 cores per plot ×6 plots). Sequence processing was performed in mothur v1.28.0 [34] and generally followed the recommendations described by Schloss *et al*. [35]. Raw sequences (598,730 reads) were denoised with PyroNoise [36] and then sequences with mismatches to forward primer or barcode sequences, ambiguous bases, and homopolymer stretches greater than 8 bp were removed. Barcodes and primer sequences were removed, short sequences were discarded, and the remaining sequences were trimmed to exactly 250 bp. The denoised and quality filtered sequences were then aligned to the Silva reference alignment (v102) [37]. Preclustering of aligned sequences was employed using an abundance weighted single-linkage preclustering step at ∼2% difference (maximum 2 bp difference over 250 bp) [38]. Chimeras were identified and removed using the UChime algorithm [39] and sequences were classified with an 80% bootstrap cutoff to the Ribosomal Database Project (RDP) 16S rRNA reference database Release 9 [40] using the RDP Classifier [41]. Sequences classified as *Archaea*, *Eukaryota*, chloroplasts, mitochondria, and unclassified sequences were removed (<0.01% of sequences), leaving only sequences classified as *Bacteria*. Pairwise distances were calculated on aligned sequences and then sequences were clustered into operational taxonomic units (OTUs) at 3% difference using the average neighbor method. The taxonomy of each OTU was assigned in mothur as the consensus taxonomy for at least 51% of the sequences within the OTU.

Sequence processing was performed with technical replicates separated (60 samples) and then with technical replicates pooled (30 samples). Sequences were subsampled to either 5,800 sequences (technical replicates separated) or 9,800 sequences (technical replicates pooled), to allow comparisons between samples with different sequencing depth. Beta diversity analysis using NMDS ordination of Bray-Curtis distances demonstrated that technical replicates clustered together and thus had similar bacterial communities (Figure S1). The mean Bray-Curtis dissimilarity between pairs of technical replicates was 0.21±0.06 (standard deviation) while the mean dissimilarity between all pairs of samples within each site was 0.39±0.05. This result indicates that the soil core homogenization was sufficient, because replicate soil samples from the same core gave reproducible bacterial communities. Thus, each sequenced soil sample is representative of the entire soil core. Therefore, technical replicates were pooled for all further data analysis. The final quality-filtered and subsampled dataset contained 294,000 sequences with 30 samples and 9,800 sequences per sample.

### Statistical analyses

Comparisons of soil parameters across plots were made using one-way analysis-of-variance (ANOVA) in R [42]. Bacterial diversity measures including the Inverse Simpson's Index and Bray-Curtis dissimilarity matrices were calculated on 3% OTUs in mothur [34]. Relationships between bacterial diversity and soil chemical data were investigated by plotting and with the function cor.test in R. Nonmetric multidimensional scaling (NMDS) ordinations on Bray-Curtis dissimilarities, permuted analysis of variance (PERMANOVA), and permuted analysis of multivariate dispersions were performed using the functions metaMDS, adonis, and betadisper from the vegan package in R [43], [44]. Because of the large differences between forests, including elevation, climate, and sampling date, the forests were treated as three separate experiments for pairwise comparisons of disturbance levels by PERMANOVA. Canonical analysis of principal coordinates (CAP) implemented through the capscale function in vegan was used to perform constrained ordination to test for linear relationships between bacterial community composition (Bray-Curtis dissimilarities) and soil chemical parameters within each forest. All permutation tests were performed with 999 permutations. Figures were prepared with R and the package ggplot2 [45].

## Results and Discussion

### Botanical diversity

The botanical diversity of the six plots is summarized in Table 2. At Analalava and Vohibe, the plots in less disturbed forest had more vascular plant morphospecies than those in more disturbed forest. The reverse was true for the plots at Pointe à Larrée, with the more disturbed plot having higher plant species richness. The moderately disturbed plot at Pointe à Larrée included much lower diversity of vascular plants than the moderately disturbed plot at Analalava or the little disturbed plot at Vohibe. The total stem DBH and stem abundance in various size classes showed a striking structural difference between the little disturbed plot at Vohibe and its very disturbed counterpart. The plots at Analava and Pointe à Larrée also had a generally consistent decrease in stem DBH and stem abundance with disturbance, but the differences between the disturbance levels much less pronounced. While the little disturbed forest plot at Vohibe included more large trees and a much higher total stem basal area than the two plots at Analalava, both of the latter included a morphospecies richness of vascular plants similar to that of the little disturbed plot at Vohibe.

### Bacterial community composition

**Figure 1:**
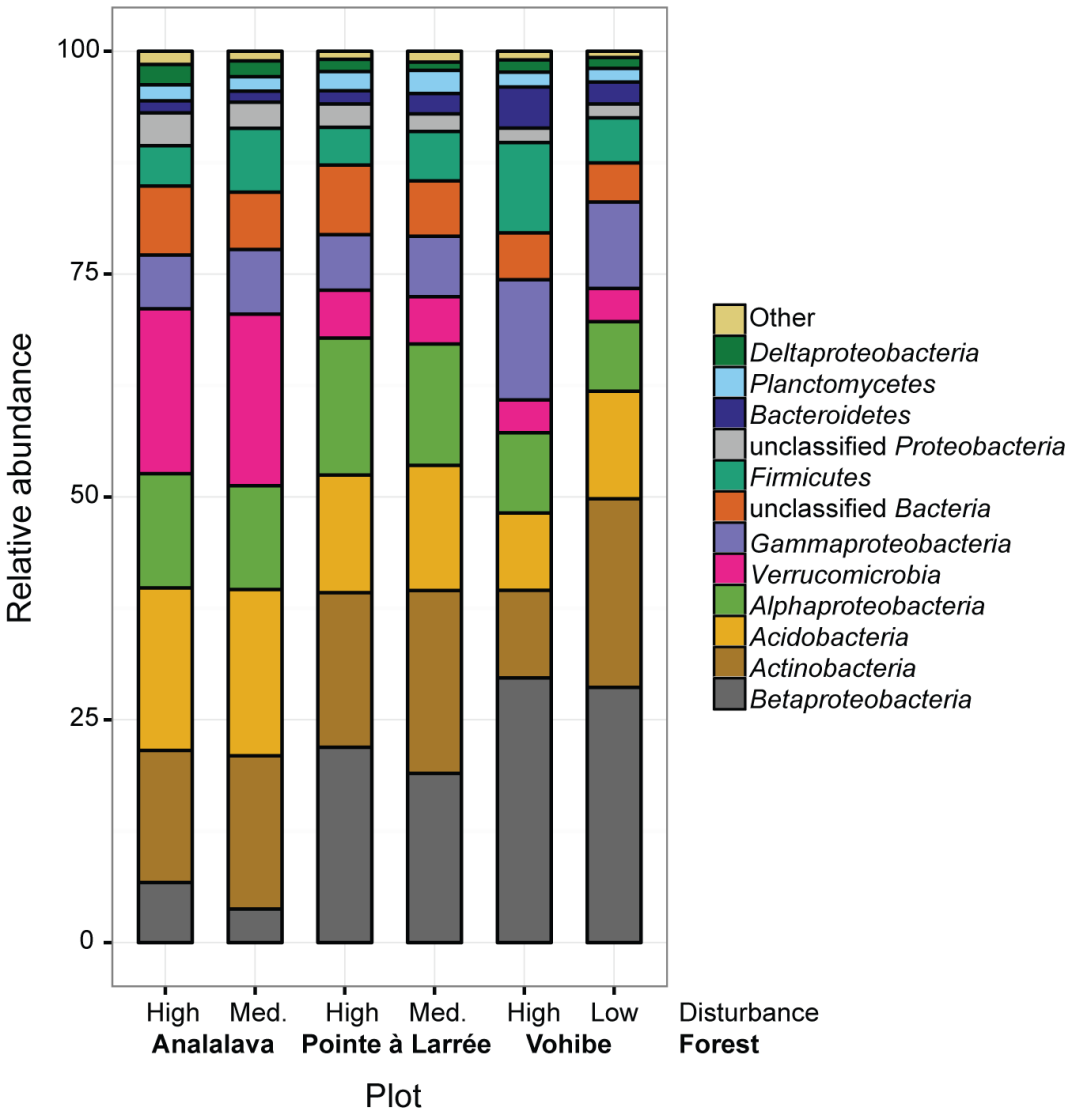
Average relative abundance of dominant bacterial taxa by plot. Sequences in 3% OTUs were classified and grouped at the phylum level, with *Proteobacteria* further divided by class. Taxa representing <1% of the total sequences were grouped as Other.

**Figure 2:**
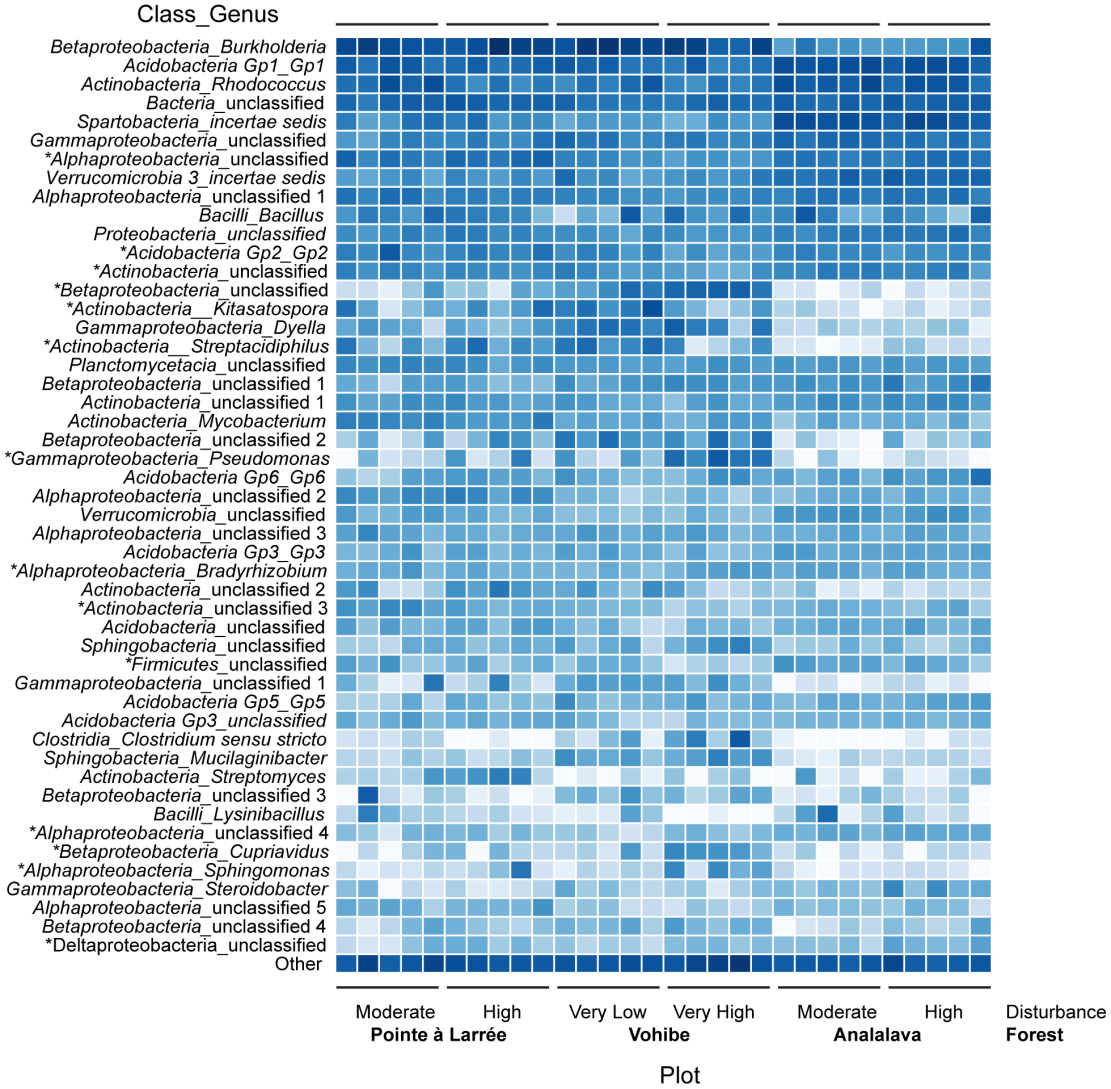
Log transformed abundance of dominant bacterial genera by sample. Sequences in 3% OTUs were classified and grouped at the genus level. Taxa representing <0.3% of the total sequences were grouped as Other. Darker color indicates higher relative abundance. Asterisks indicate taxa with significant shifts in abundance between disturbance levels at Vohibe (see Table 5).

In order to compare the relative abundance of bacterial taxonomic groups observed in this study, 3% OTUs were classified according to the RDP taxonomy and grouped at the phylum level, except for proteobacterial groups, which were further divided by class (Figure 1). The most abundant bacterial group in the Pointe à Larrée and Vohibe plots was *Betaproteobacteria*, representing an average of about 20–30% of sequences within each plot. In contrast, the Analalava plots contained mostly *Acidobacteria* and *Verrucomicrobia*. Other abundant groups with an average of >5% of sequences across all plots were *Actinobacteria*, *Alphaproteobacteria*, *Gammaproteobacteria*, and *Firmicutes*. The bacterial community composition was further analyzed by grouping OTUs at the genus level (Figure 2). Abundant genera across all soil samples included *Burkholderia*, the *Acidobacteria* Group 1, *Rhodococcus*, and *Bacillus*. The functional and ecological roles of the *Acidobacteria* remain poorly characterized, but there is evidence for strong correlations between abundance of acidobacterial groups and pH [8], [46], as well as indications that many *Acidobacteria* may be adapted to an oligotrophic lifestyle [47]. Naether *et al*. observed that the Group 1 *Acidobacteria* were dominant in temperate forest soils, while the Group 6 Acidobacteria were most prevalent in grassland soils [46]. The abundance of the betaproteobacterial genus *Burkholderia* observed here is consistent with the recent identification of this genus as an acid tolerant group that may outcompete other taxa in acidic soils such as those in our study [48]. A BLAST analysis of a representative sequence from the dominant *Burkholderia* OTU in our dataset showed that the closest relative was *Burkholderia sabiae* strain STM7319, which clusters within the plant-beneficial-environmental (PBE) group of *Burkholderia* [49]. The next two most abundant *Burkholderia* OTUs in our dataset also clustered with this group rather than the typically pathogenic *Burkholderia cepacia* complex. Members of the PBE clade are often associated with plants and may be capable of nitrogen fixation or otherwise promote plant growth. Amplicon-based community profiling has many well-documented limitations, including DNA extraction and primer bias, which constrain our ability to draw conclusions about absolute abundance of bacterial groups. However, despite any bias, sequencing results are reproducible, so comparisons can be made concerning relative abundance of taxa in samples with consistent DNA extraction and sequencing methods [50].

### Bacterial diversity and disturbance

The Inverse Simpson diversity index is a measure of alpha diversity that accounts for the richness and evenness of OTUs observed within a single sample. We observed a trend of lower bacterial diversity in the less disturbed plots (Table 3, Figure S2), although this difference was not significant. We observed no significant difference in the Inverse Simpson index between each of the plots (treating each of the six plots separately) or between each of the forests (combining the two plots in each forest). Comparisons made using richness estimators (number of OTUs, Chao1) and a phylogenetic diversity measure (Faith's PD) gave similar results (data not shown). Comparisons using Smith and Wilson's metric of evenness demonstrated a significant difference at the level of plots (*p* = 0.002) and forests (*p* = 0.008). A Tukey's honestly significant difference (HSD) test showed that the high impact plot at Pointe à Larrée had significantly lower evenness than the low impact plot at Vohibe (*p* = 0.003) and the moderate and high impact plots at Analalava (*p* = 0.002 and 0.04 respectively). However, there was no significant difference in evenness between any of the pairs of plots within each forest (*p*>0.05).

**Table 3:** Bacterial diversity statistics^a^ calculated at the OTU (3%) level.

Plot	Good's coverage %	# OTUs	Inverse Simpson	Shannon	Smith and Wilson's Evenness
A1	95.8 (0.9)	970 (178)	51.0 (23.3)	5.08 (0.36)	0.34 (0.02)
A2	96.5 (0.5)	819 (78)	37.1 (10.0)	4.77 (0.17)	0.37 (0.01)
P1	94.0 (0.9)	1212 (174)	40.0 (25.1)	5.12 (0.47)	0.29 (0.03)
P2	95.7 (0.9)	972 (199)	34.0 (13.6)	4.88 (0.42)	0.33 (0.04)
V1	94.9 (0.8)	1031 (186)	43.1 (14.7)	4.96 (0.37)	0.33 (0.03)
V2	96.0 (0.8)	838 (120)	29.1 (14.6)	4.64 (0.32)	0.37 (0.02)

a Shown are the mean and (sd) of each statistic calculated for 5 sequencing samples per plot.

These results indicate that disturbance from selective timber harvesting or historical cultivation did not significantly affect the soil bacterial diversity, although there was a slight trend towards higher bacterial diversity with disturbance in all forests. We did not detect a significant difference in bacterial diversity or richness even between the Vohibe plots where we observed dramatic differences in plant species richness and vegetation structure. Other studies have found correlations in bacterial diversity with land-use, with agricultural sites having higher diversity than forested sites in tropical soils, but these sites also differed in soil environmental parameters [6], [13]. The results of our study more closely resemble those of Suleiman *et al.* in the Brazilian Pampa, where no significant difference in alpha diversity was found between eight-year-old de-forested pasture as compared to surrounding undisturbed forest [12]. While we did not find significant effects of land-use on soil bacterial richness at the resolution afforded by the 16S rRNA gene, there could be finer level changes in the diversity of functional genes or natural product biosynthetic genes [51]. It remains an open question whether bacterial taxonomic diversity (richness) correlates with ecosystem function, and if loss of microbial diversity affects community resilience or function [21], [52], [53]. Moreover, a recent study found large fluctuations in soil bacterial diversity over time, with temporal variability exceeding that attributed to land-use [53]. More sampling replicates obtained from different seasons might be required to reveal differences in bacterial diversity with disturbance level or botanical diversity.

### Bacterial diversity and soil properties

**Figure 3:**
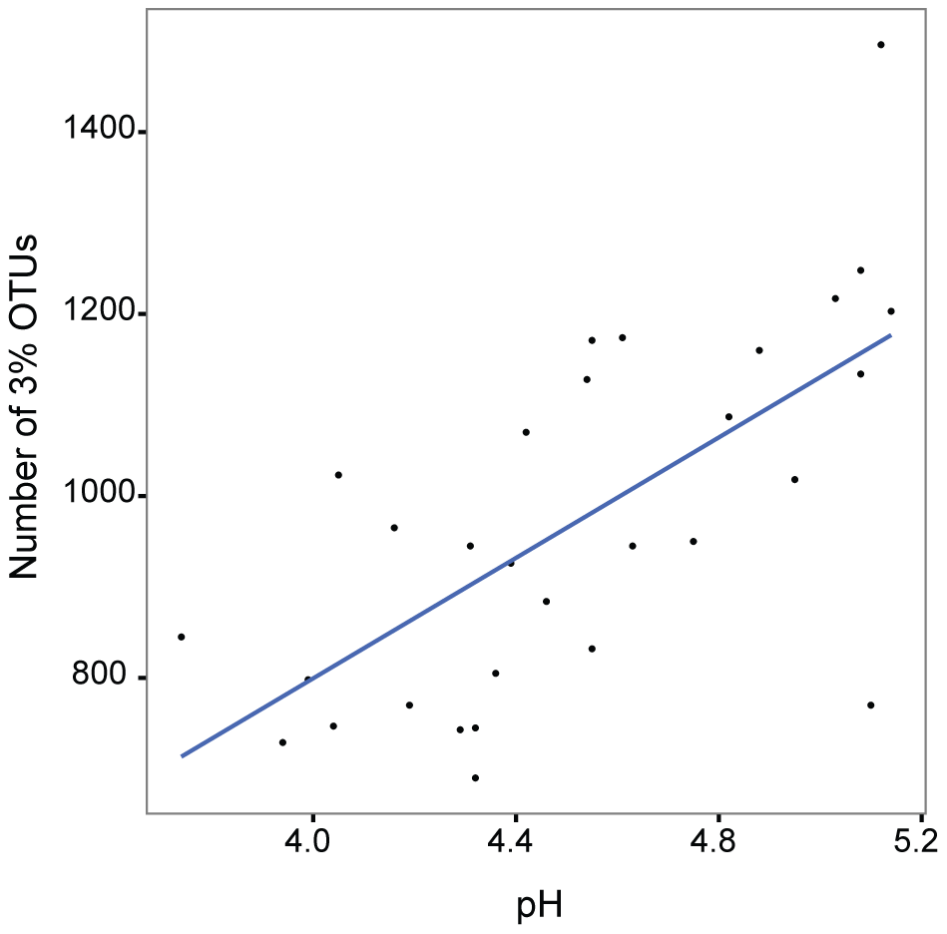
Richness and soil pH. Bacterial community richness is shown as the number of 3% OTUs. Pearson's *r* = 0.66, *p*<0.0001.

Several studies have documented a strong relationship between bacterial richness and soil pH at both global and local scales [5], [6], [8]. Although we did not observe site-dependent changes in diversity with pH, we hypothesized that changes in soil properties irrespective of site might be related to bacterial community richness. In fact, when looking at all samples collectively, the number of OTUs per sample was positively correlated with soil pH (Pearson's *r* = 0.66, *p*<0.0001), which varied from 3.7–5.1 in our study (Figure 3). Measures of diversity, including the Inverse Simpson's Index, were also positively correlated with soil pH across all samples. This relationship was significant but weaker than that observed by Rousk *et al.* at a single site, a gradient of pH 4–8 at the Hoosfield acid strip long-term agricultural research site in Harpenden, UK [8]. We found no significant correlations between richness and the other measured soil parameters including organic C, total N, or C:N ratio.

### Bacterial community composition varies with forest more than with disturbance level

**Figure 4:**
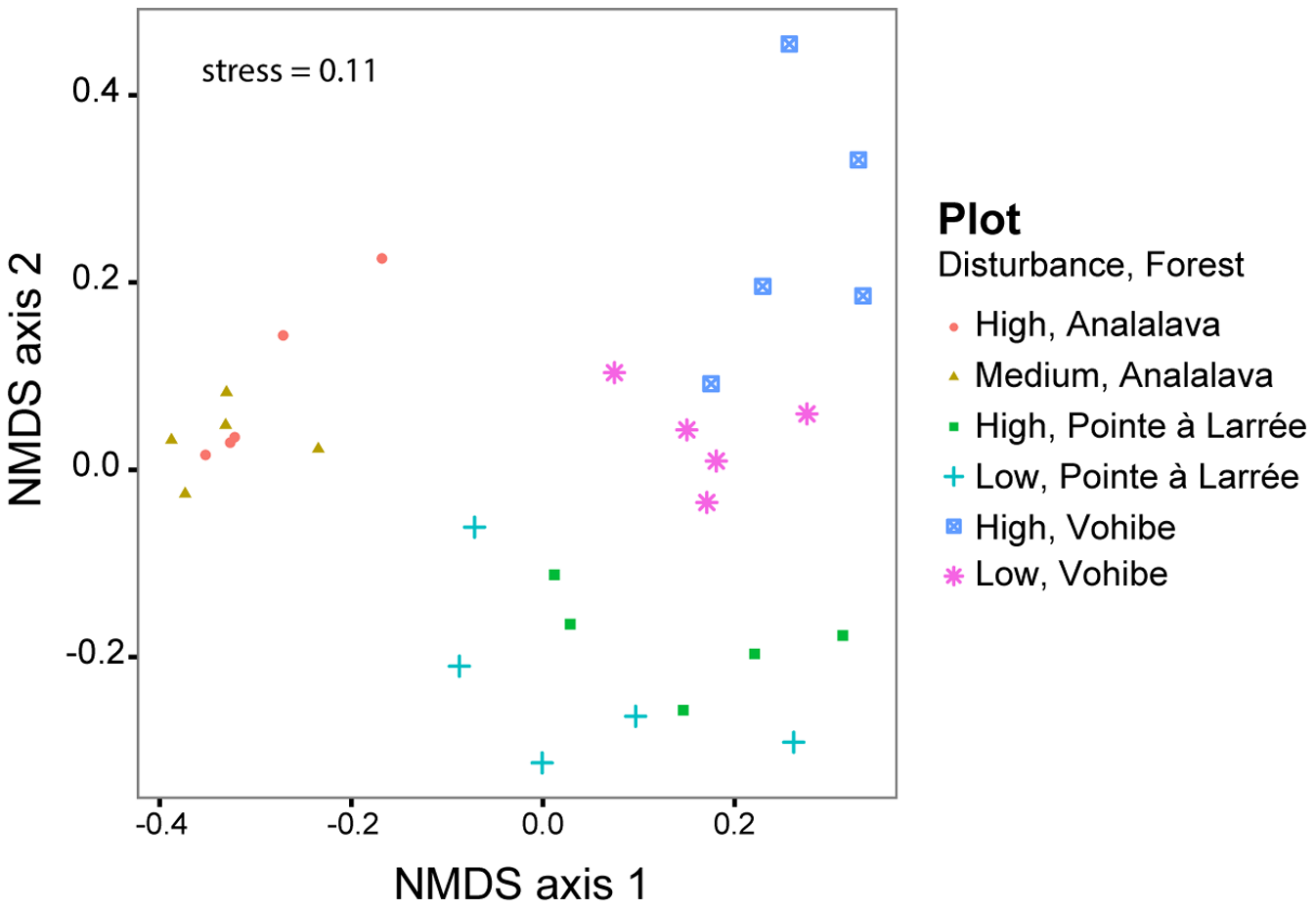
Nonmetric multidimensional scaling (NMDS) plot of pairwise Bray-Curtis dissimilarities between bacterial communities in soil calculated with 3% OTUs.

An NMDS ordination of Bray-Curtis dissimilarities showed strong clustering of bacterial communities by forest as compared to levels of disturbance (Figure 4). The significance of this clustering was tested using a nested PERMANOVA design (sites nested within forests) with Bray-Curtis dissimilarities calculated on 3% OTUs. We observed significant differences between bacterial community compositions at the three forest sites (pseudo-*F*  = 10.47, *p* = 0.001) and a significant interaction effect between forests and sites (pseudo-*F* 1.87, *p* = 0.009). Pairwise comparisons between forests also showed strong differences in the bacterial community structures (A-V: *t* = 12.95; A-P: *t* = 11.20; V-P: *t* = 5.44, by PERMANOVA, Bonferroni corrected *p* = 0.003 for these pairwise comparisons). The samples from Analalava had significantly lower spread than those from Pointe à Larrée, but no other significant differences were detected in the dispersion of samples within forests (pairwise comparisons with betadisper, A-V: *t* = 3.80, *p* = 0.20; A-P: *t* = 7.03, *p* = 0.02; V-P: *t* = 0.065, *p* = 2.41, with Bonferroni correction).

Pairwise PERMANOVAs were then performed between the impact levels within each forest in order to test whether soil bacterial community composition varied with the level of disturbance. Only the bacterial communities at Vohibe varied significantly with disturbance level (*t* = 3.0, *p* = 0.007). The NMDS ordination and smaller PERMANOVA pseudo-*F*/*t* statistic indicate that the difference between the bacterial communities of the two sites at Vohibe is less than that observed between forests. Pairwise tests for homogeneity of group variance between the impact levels within each forest were not significant (*p*>0.05 for all pairwise comparisons). The two Vohibe sites were the most different in terms of impact level (very high vs. very low impact), as indicated by the measured differences in plant morphospecies richness and tree cover (Table 2). It is therefore perhaps not surprising that we were able to observe a difference in bacterial structure between this pair of plots. By contrast, no areas representing a low level of disturbance were available at Analalava and Pointe à Larrée, and so the selected pairs of sites represent moderate and high levels of impact from selective harvesting of trees. To test for differences in soil parameters between plots, we used one-way ANOVAs followed by Tukey's HSD test, and the only significant differences between paired plots within each forest were in pH at Vohibe and in total N at Pointe à Larrée. The pH levels of the soil samples from the high disturbance Vohibe forest were higher compared to those of the low disturbance plot, perhaps resulting from the history of burning followed by shifting cultivation in the disturbed plot. Thus, the observed change in bacterial community structure between sites with different impact levels at Vohibe is correlated with differences in botanical diversity and with a change in soil pH.

### Beta diversity and soil properties

**Figure 5:**
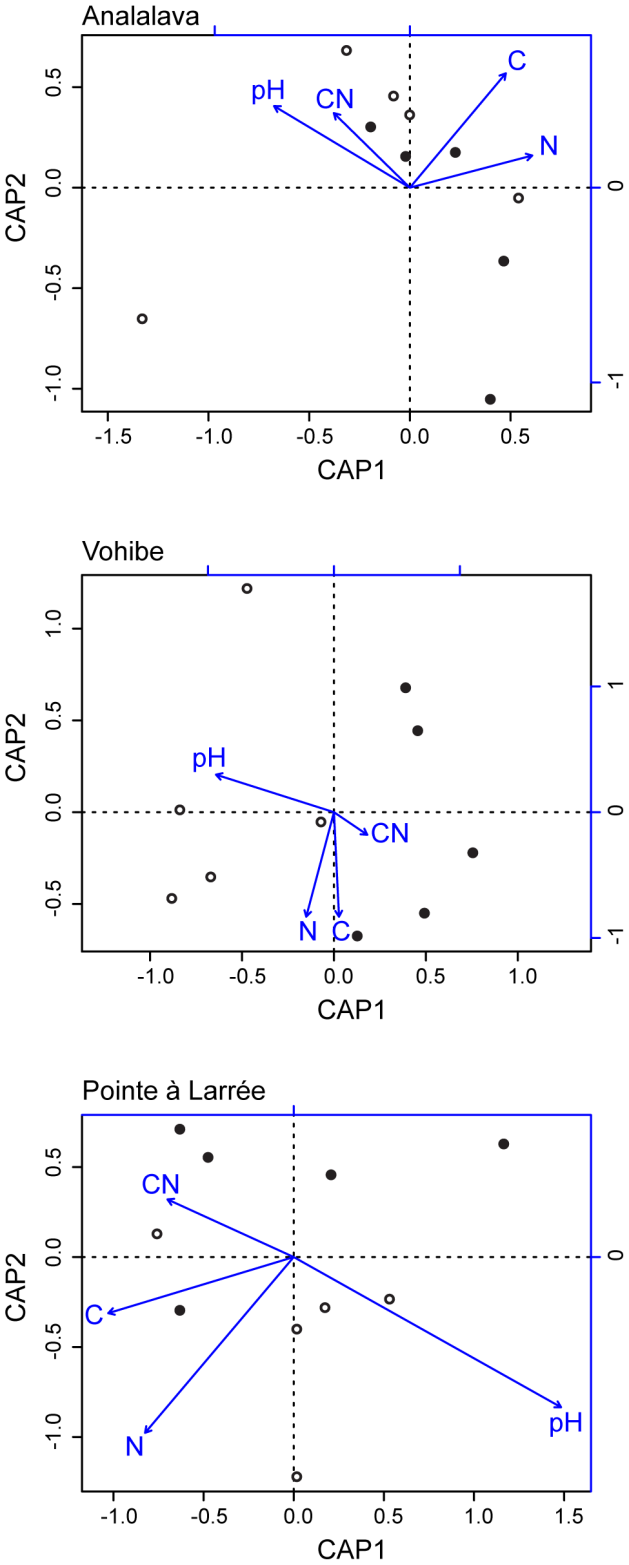
CAP ordination biplots of the bacterial community composition data and soil chemical properties (arrows) within each forest. Closed circles indicate sites from the lower disturbance plot and open circles indicate sites from the higher disturbance plot.

We expected that soil samples with more similar soil chemistry would also host more similar bacterial communities. To test this hypothesis, CAP ordinations of the community composition data (relative abundance of 3% OTUs) constrained by the soil chemical data were performed within each forest (Figure 5). Within all forests, pH was significantly correlated with bacterial community structure (*p*<0.05 for all tests). The total N was only significant within the Pointe à Larrée forest. Total C and CN were not significant within any forest and C tended to covary with N. As expected given the previous unconstrained ordination, pH was significantly aligned with CAP axis 1 and the two disturbance sites at Vohibe also separate along this axis. Past studies have demonstrated associations of bacterial community composition with pH, C, N, and/or C:N ratio, depending on the sample type or treatment [6], [11], [24]. Fierer *et al*. [9] also observed a correlation between bacterial community phylogenetic distances and high soil N amendment levels in temperate agricultural soils. Strong shifts in soil bacterial community composition have been reported with nitrogen amendment to soil samples and year-long incubation under controlled, laboratory conditions [54]. While pH is strongly correlated with bacterial community composition in the majority of soil bacterial community studies, the importance of other soil edaphic properties may vary depending on the soil type or treatment.

### Differentially abundant groups between plots at Vohibe

The program Metastats [55] was used to determine which bacterial groups were differentially abundant between the Vohibe plots and therefore contributed to the significant difference in bacterial community composition between these plots. At the OTU level, this analysis detected greater than 200 bacterial groups with significant changes in abundance (data not shown). In order to relate these changes to classified bacterial taxa, the Metastats analysis was performed with OTUs grouped at the class and genus levels of the RDP taxonomy (Table 4 and Table 5). Differences in abundance of bacterial taxa were considered significant with *p*<0.05. At the class level, the relative abundance of bacterial groups *Acidobacteria* Group 2, unclassified *Firmicutes*, and *Actinobacteria* were all lower in the high impact Vohibe plot as compared to the low impact plot (Table 4). Conversely, the abundance of class *Opitutae* within the phylum *Verrucomicrobia* was higher in the high impact plot compared to the low impact plot samples from Vohibe. Changes in acidobacterial groups with pH have been well documented. For example, Rousk *et al.* [8] also observed a decrease in the relative abundance *Acidobacteria* Gp 2 with increasing pH in temperate agricultural soil. In the same study, no consistent trend in the abundance of *Actinobacteria* was observed from pH 4–8, but within the relevant pH range of our samples (∼4–5), Rousk *et al.* did observe a decrease in actinobacterial abundance. At a larger geographic scale, Fierer *et al.* [9] found that the abundance of the phylum *Acidobacteria* decreased with increasing pH, while the relative abundance of *Actinobacteria* increased [56]. However, the increasing abundance of *Actinobacteria* was observed mainly between pH 5–7. *Verrucomicrobia* were not dominant groups in these studies, perhaps because of primer bias, and so differential abundances were not reported.

**Table 4:** Bacterial phylotypes at the Class level with differing abundance between Vohibe high impact (V1) and low impact (V2) sites (Metastats *p*<0.05).

RDP Classification	V1 rel. abundance (%)	V2 rel. abundance (%)	Abundance at higher impact plot
Phylum_Class	mean (sd)	mean (sd)	
*Firmicutes*_unclassified	0.14 (0.04)	0.46 (0.10)	lower
*Acidobacteria*_*Gp2*	1.1 (0.2)	2.3 (0.3)	lower
*Verrucomicrobia*_*Opitutae*	0.09 (0.02)	0.03 (0.02)	higher
*Actinobacteria*_*Actinobacteria*	9.8 (1.6)	21.2 (4.5)	lower

**Table 5:** Bacterial phylotypes at the Genus level with differing abundance between Vohibe high impact (V1) and low impact (V2) sites (Metastats *p*<0.05).

RDP Classification	V1 rel. abundance (%)	V2 rel. abundance (%)	Abundance at higher impact plot
Class_Genus	mean (sd)	mean (sd)	
*Actinobacteria_Solirubrobacter*	0.00008 (0.00002)	0.00029 (0.00004)	lower
*Deltaproteobacteria_*unclassified	0.0037 (0.0004)	0.0016 (0.0004)	higher
*Gammaproteobacteria_Pseudomonas*	0.05 (0.01)	0.006 (0.003)	higher
*Actinobacteria_*unclassified	0.0023 (0.0005)	0.0045 (0.0003)	lower
*Alphaproteobacteria_Bradyrhizobium*	0.010 (0.001)	0.0044 (0.0006)	higher
*Betaproteobacteria_*unclassified	0.061 (0.006)	0.026 (0.009)	higher
*Firmicutes_*unclassified	0.001 (0.003)	0.0045 (0.0009)	lower
*Alphaproteobacteria_*unclassified	0.0039 (0.0006)	0.0014 (0.0004)	higher
*Betaproteobacteria_Cupriavidus*	0.014 (0.003)	0.003 (0.002)	higher
*Acidobacteria Gp2_Gp2*	0.011 (0.002)	0.023 (0.003)	lower
*Alphaproteobacteria_*unclassified	0.013 (0.001)	0.021 (0.003)	lower
*Actinobacteria_Streptacidiphilus*	0.010 (0.005)	0.036 (0.008)	lower
*Planctomycetacia_Gemmata*	0.00008 (0.00004)	0.0006 (0.0002)	lower
*Gammaproteobacteria_Coxiella*	0.00014 (0.00005)	0	higher
*Gammaproteobacteria_*unclassified	0.0007 (0.0002)	0.00002 (0.00002)	higher
*Actinobacteria_Nocardioides*	0.0018 (0.0007)	0.00002 (0.00002)	higher
*Caldilineae_Caldilinea*	0.00006 (0.00002)	0	higher
*Betaproteobacteria_Georgfuchsia*	0.00006 (0.00002)	0	higher
*Alphaproteobacteria_Sphingomonas*	0.012 (0.004)	0.0008 (0.0002)	higher
*Sphingobacteria_Ferruginibacter*	0.0010 (0.0004)	0.0024 (0.0005)	lower
*Anaerolineae_*unclassified	0.00010 (0.00005)	0	higher
*Actinobacteria_Kitasatospora*	0.007 (0.003)	0.05 (0.02)	lower
*Acidobacteria Gp22_Gp22*	0.00008 (0.00004)	0	higher
*Alphaproteobacteria_Hyphomicrobium*	0.00008 (0.00004)	0	higher
*Gammaproteobacteria_Salmonella*	0.00008 (0.00004)	0	higher
*Planctomycetacia_Planctomyces*	0.00016 (0.00005)	0.00004 (0.00002)	higher
*Actinobacteria_*unclassified	0.010 (0.002)	0.018 (0.004)	lower

When the shifts in the bacterial community composition at Vohibe were examined in greater detail at the genus level, we also observed higher abundance of the genus *Bradyrhizobium* and of an unclassified group within the *Oxalobacteraceae* in the high impact plot; both of these clades contain nitrogen-fixing members (Table 5). Interestingly, several bacterial genera known for their roles as human and animal pathogens had significantly higher abundance with disturbance, including *Pseudomonas*, *Coxiella*, and *Salmonella*. This shift might reflect the history of shifting cultivation in the higher impact site at Vohibe. Corroborating the results at the class level, the abundance of several actinobacterial genera, including *Solirubrobacter*, *Streptacidiphilus*, and *Kitasatospora* as well as unclassified *Actinobacteria*, were lower in the high impact plot (Table 5 and Figure S3). Only one actinobacterial genus, the *Nocardioides*, was observed in significantly higher abundance in the higher impact plot.

Members of the phylum *Actinobacteria* are well known for their role in degrading complex organic matter in soils and for their potential as producers of bioactive natural products. The significant decrease in the relative abundance of *Actinobacteria* amplicons in the disturbed plot at Vohibe indicates that sampling across disturbance levels could improve the sampled biodiversity for this natural product discovery target phylum. Similarily, Zabinski & Gannon [22] showed that disturbance from recreational camping decreased the percentage of culturable bacteria with *Actinomycete*-like morphology in subalpine soils. However, the sequencing survey performed in this study measures relative abundance of amplicons and cannot distinguish between true shifts in the numbers of *Actinobacteria* and changes in abundance due to shifts in other groups. The results from our work suggest that disturbance from shifting cultivation at Vohibe could decrease the proportion of *Actinobacteria,* and we plan future experiments to test this hypothesis directly using comparisons of culturable bacterial isolates, direct sequencing, and characterization of the diversity of natural product biosynthetic genes.

## Conclusions

Contrary to our initial hypothesis, we found no significant differences in soil bacterial diversity with different disturbance levels, although there was a non-significant trend towards higher richness with disturbance. Comparison of technical replicates from each bulk soil core demonstrated that the observed bacterial community composition within samples from the same homogenized core were reproducible. We detected a significant change in soil bacterial community composition between the low and high disturbance plots in Vohibe forest, but not at Analalava or Pointe à Larrée where there was less difference between disturbance levels in the plots sampled. The Vohibe sites exhibited a large decrease in woody plant morphospecies richness and a decrease in tree cover in the high disturbance plot compared to relatively intact, native forest in the low disturbance plot. The change in bacterial community composition with disturbance at Vohibe was also correlated with an increase in soil pH, which may have resulted from historical burning during shifting cultivation. The study design did not allow us to differentiate between co-varying parameters and so future experiments will be required to distinguish direct and indirect effects. Future work might also look for shifts in microbial functional genes that could be masked by this OTU level taxonomic analysis. Our study focused on the soil bacterial diversity, but changes in botanical diversity and land use would also be expected to influence archaeal and fungal taxa that are known to serve important roles within soil microbial communities. The relative changes in bacterial community composition between sites were much greater than between disturbance levels, suggesting that widespread geographic sampling across forests would maximize access to bacterial diversity rather than collecting multiple samples from different disturbance levels within a given forest.

## Supporting Information

Figure S1 **Nonmetric multidimensional scaling (NMDS) plot of pairwise Bray-Curtis distances between soil samples calculated with 3% OTUs.** Technical replicates (two DNA samples from the same homogenized bulk soil core) are labeled with the same number.
(TIF)

Figure S2 **Mean bacterial alpha diversity in each plot as measured by the Inverse Simpson's index of diversity.** Errors bars represent the standard error of the mean for five soil samples.
(TIF)

Figure S3 **Average relative abundance of actinobacterial genera by plot. Sequences in 3% OTUs were classified and grouped at the genus level.** Taxa representing <0.1% of the total sequences were grouped as Other.
(TIF)

Table S1 **Chemical data for homogenized soil cores.**
(DOCX)
