# Supplementary figures and images for: Bacterial Communities in Malagasy Soils with Differing Levels of Disturbance Affecting Botanical Diversity
Source: PLoS One. 2014 Jan 20;9(1):e85097. doi: 10.1371/journal.pone.0085097 (PMC3896373; doi:10.1371/journal.pone.0085097)

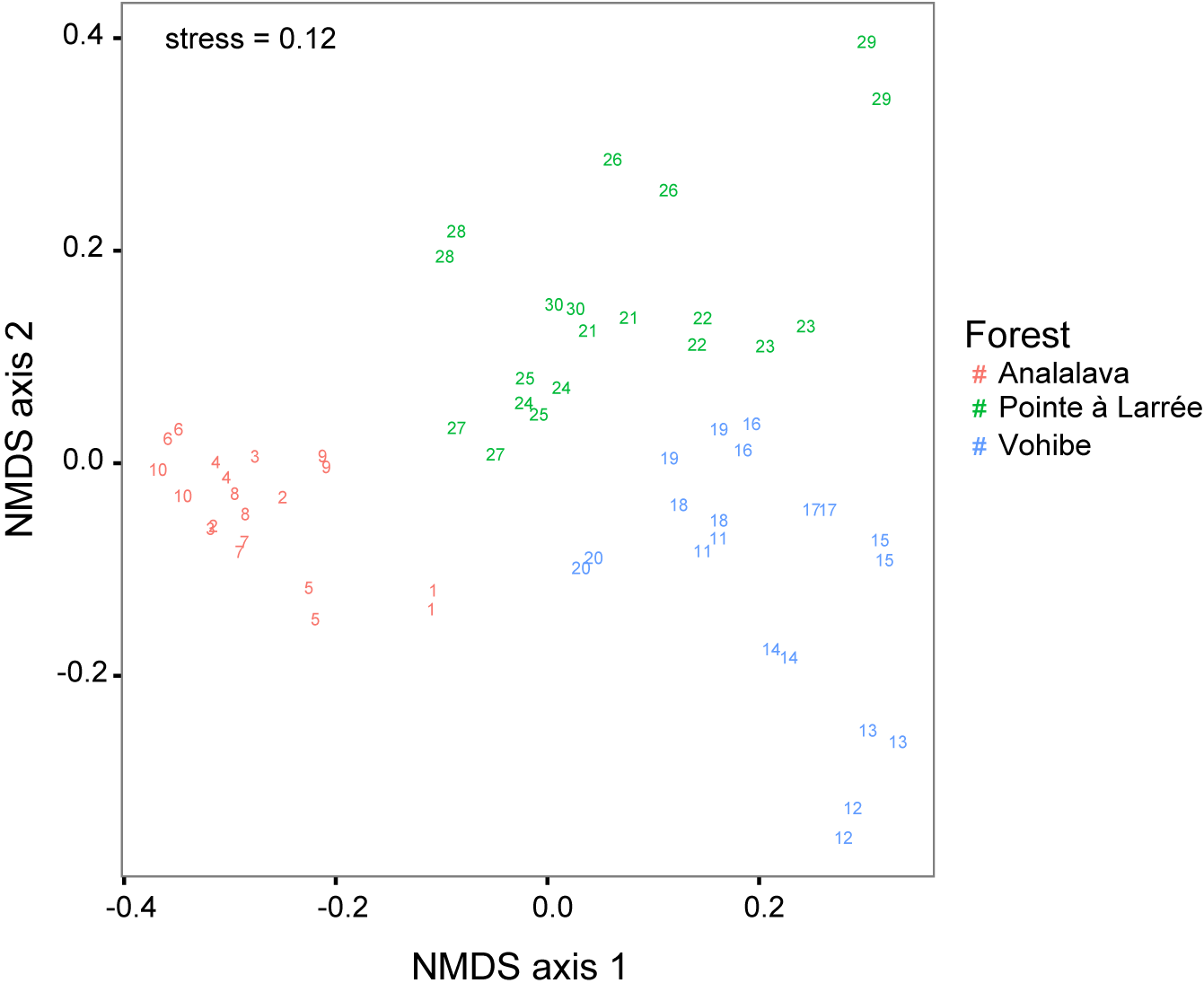

Supplement: Figure S1 — Nonmetric multidimensional scaling (NMDS) plot of pairwise Bray-Curtis distances between soil samples calculated with 3% OTUs. Technical replicates (two DNA samples from the same homogenized bulk soil core) are labeled with the same number. (TIF) [file pone.0085097.s001.tif]

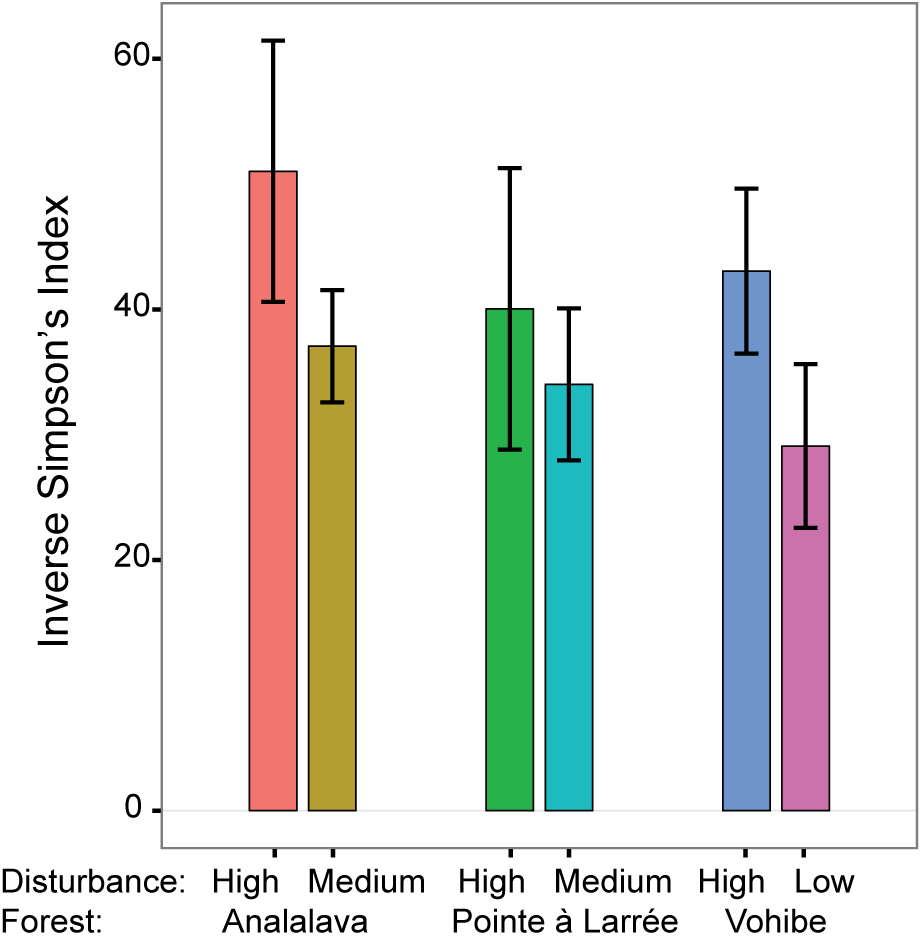

Supplement: Figure S2 — Mean bacterial alpha diversity in each plot as measured by the Inverse Simpson's index of diversity. Errors bars represent the standard error of the mean for five soil samples. (TIF) [file pone.0085097.s002.tif]

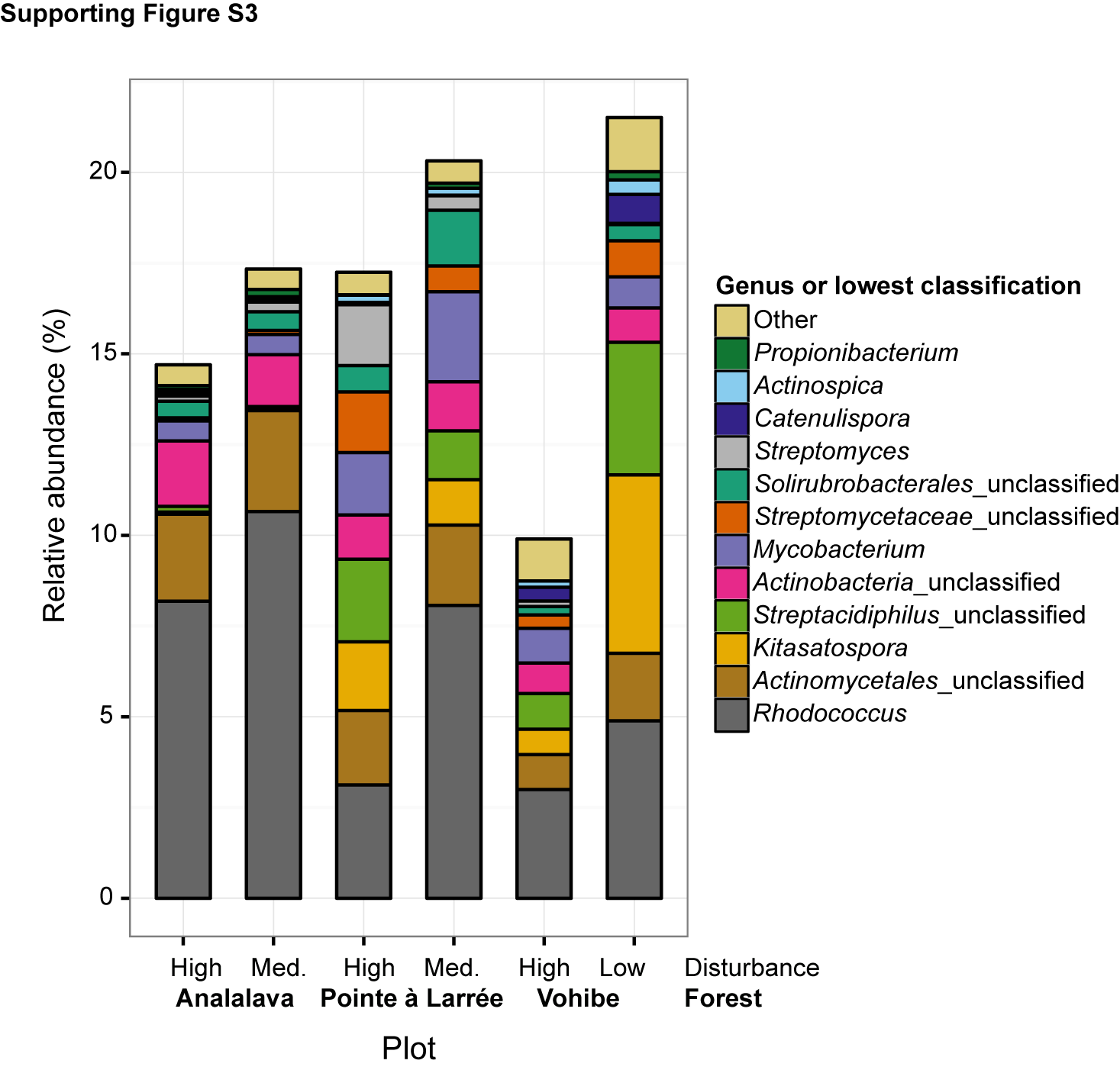

Supplement: Figure S3 — Average relative abundance of actinobacterial genera by plot. Sequences in 3% OTUs were classified and grouped at the genus level. Taxa representing <0.1% of the total sequences were grouped as Other. (TIF) [file pone.0085097.s003.tif]
