# Supplementary material for: Bacterial Communities in Malagasy Soils with Differing Levels of Disturbance Affecting Botanical Diversity
Source: PLoS One. 2014 Jan 20;9(1):e85097. doi: 10.1371/journal.pone.0085097 (PMC3896373; doi:10.1371/journal.pone.0085097)
Supplement: Table S1 — Chemical data for homogenized soil cores. (DOCX) [file pone.0085097.s004.docx]

Supporting Table S1. Chemical data for homogenized soil cores.

| Sample | Plot | Soil pH | Total N (%) | Total Organic C (%) | C:N ratio |
| --- | --- | --- | --- | --- | --- |
| AD1 | A1 | 4.54 | 0.11 | 2.12 | 20.19 |
| AD2 | A1 | 4.39 | 0.15 | 2.57 | 17.48 |
| AD3 | A1 | 4.46 | 0.18 | 3.50 | 20.00 |
| AD4 | A1 | 4.29 | 0.19 | 3.33 | 17.62 |
| AD5 | A1 | 4.55 | 0.18 | 3.60 | 20.57 |
| AN1 | A2 | 4.19 | 0.17 | 3.42 | 20.36 |
| AN2 | A2 | 4.63 | 0.22 | 3.57 | 16.45 |
| AN3 | A2 | 4.36 | 0.18 | 3.20 | 18.29 |
| AN4 | A2 | 4.55 | 0.21 | 3.44 | 16.38 |
| AN5 | A2 | 4.32 | 0.20 | 2.75 | 13.55 |
| AmbD1 | V1 | 4.75 | 0.36 | 3.38 | 9.29 |
| AmbD2 | V1 | 5.03 | 0.34 | 4.74 | 14.11 |
| AmbD3 | V1 | 5.14 | 0.19 | 2.95 | 15.61 |
| AmbD4 | V1 | 4.95 | 0.31 | 5.52 | 17.92 |
| AmbD5 | V1 | 5.10 | 0.29 | 4.41 | 15.37 |
| AmbN1 | V2 | 4.32 | 0.22 | 3.45 | 15.90 |
| AmbN2 | V2 | 4.04 | 0.30 | 6.16 | 20.47 |
| AmbN3 | V2 | 3.74 | 0.27 | 4.35 | 15.88 |
| AmbN4 | V2 | 4.31 | 0.25 | 4.16 | 16.98 |
| AmbN5 | V2 | 4.16 | 0.32 | 5.43 | 17.24 |
| PD1 | P1 | 4.42 | 0.67 | 22.72 | 34.17 |
| PD2 | P1 | 5.08 | 0.47 | 6.56 | 13.99 |
| PD3 | P1 | 4.82 | 0.37 | 5.39 | 14.53 |
| PD4 | P1 | 4.88 | 0.36 | 6.56 | 18.38 |
| PD5 | P1 | 5.12 | 0.34 | 6.81 | 20.27 |
| PN1 | P2 | 5.08 | 0.07 | 1.75 | 24.65 |
| PN2 | P2 | 4.61 | 0.23 | 4.56 | 20.18 |
| PN3 | P2 | 3.94 | 0.22 | 4.18 | 18.66 |
| PN4 | P2 | 3.99 | 0.26 | 10.63 | 41.04 |
| PN5 | P2 | 4.05 | 0.22 | 4.76 | 21.94 |
